# Outcome of Presumptive Versus Rapid Diagnostic Tests-Based Management of Childhood Malaria - Pneumonia Overlap in Urban Nigeria: A Pilot Quasi-Experimental Study

**DOI:** 10.4314/ejhs.v20i3.69447

**Published:** 2010-11

**Authors:** Kingsley N Ukwaja, Olufemi B Aina, Ademola A Talabi

**Affiliations:** a Comprehensive Health Centre, Oke-Ilewo, Abeokuta, Ogun state, Nigeria; b Department of Internal Medicine, Ebonyi State University Teaching Hospital, Abakaliki, Ebonyi State, Nigeria

**Keywords:** Malaria management, malaria- pneumonia overlap, rapid diagnostic tests, safety, Nigeria

## Abstract

**Background:**

Symptoms of malaria and pneumonia overlap in African under-five children and the integrated management of childhood illness strategy require that such children be managed presumptively with both antibiotics and antimalarials. A 2003 WHO expert meeting recommended the evaluation of malaria rapid diagnostic test in the management of children with this overlap, but this has not been evaluated. Therefore, the objective of this study was to compare the clinical outcome of presumptive versus malaria rapid diagnostic test-based management of childhood malaria-pneumonia overlap in Nigeria.

**Methods:**

A pilot quasi-experimental study was conducted November 2009 through February 2010 in an urban comprehensive health centre in Ogun, South-Western Nigeria. First, 50 children with malaria-pneumonia symptom overlap were consecutively enrolled and treated presumptively with antibiotics and antimalarials irrespective of malaria test result (control arm).Then, another 50 eligible children were enrolled and treated with antibiotics with/out antimalarials based on rapid diagnostic test result (intervention arm). Primary endpoint: clinical cure at day-5. The data were analyzed using Epi Info version 3.4.1.

**Results:**

The intervention and control arms did not differ significantly regarding patient demographic and clinical characteristics. Clinical cure rate was slightly higher in children managed presumptively 49 (98%) than those managed rapid diagnostic test -based 47 (94%) (P = 0.31). However, rapid diagnostic test -based treated children had lower risk of receiving antimalarials compared to those treated presumptively (48% vs. 100%), (P = <0.001; relative risk 2.08, CI 1.56 to 2.78). No death or severe complications were recorded in either group at day-5 follow-up.

**Conclusion:**

Outcome of rapid diagnostic test-based treatment is not inferior to presumptive management in children with malaria-pneumonia symptom overlap. More extensive studies with larger sample sizes are needed.

## Introduction

Because of the symptom overlap between malaria and pneumonia in African children, the World Health Organization (WHO) Integrated Management of Childhood Illness (IMCI) strategy requires that such children be managed presumptively with both antibiotics and antimalarials (1, 2). The widespread adoption of artemisinin based combination therapy antimalarials in low-resource settings and the availability of malaria rapid diagnostic tests (RDTs) have led to the modification of the malaria treatment policy by the WHO. The policy recommended malarial treatment only after parasitological confirmation-this recommendation had initially been restricted to older children and adults, but in the guideline's second edition (released 2^nd^ March 2010) the RDT-based policy has been extended to younger children (3).

Although the accuracy of RDTs for malaria infection has been demonstrated (4), there is limited evidence for the safety of a test -based strategy for children below five years (5, 6). Also, though the safety of not treating RDT negative children has been confirmed, such studies were conducted mainly in the context of childhood fevers (7). In 2003, a WHO consultative meeting on childhood pneumonia recommended that studies to improve the specificity of clinical overlap for malaria and pneumonia diagnosis should be undertaken, and rapid diagnostic tests for malaria should be studied to differentiate malaria from pneumonia (8). We found no study that evaluated the use of RDT in the treatment of childhood malaria - pneumonia overlap. Therefore, this study was conducted to compare the outcome of presumptively treated children having this overlap with antibiotics and antimalarials irrespective of malaria test result versus children treated with antibiotics with/out antimalarials based on RDT result in an area of high malaria transmission.

## Patients and Methods

This was a pilot quasi-experimental study conducted November 2009 through February 2010. First, November through December 2009, fifty children with malaria-pneumonia symptom overlap were consecutively enrolled and treated presumptively with antibiotics and antimalarials irrespective of malaria test results according to national/ WHO/UNICEF guidelines (control arm). Then, January through February 2010, another 50 eligible children were consecutively enrolled and treated with antibiotics with/out antimalarials based on rapid diagnostic test result (intervention arm). The primary endpoint was clinical cure at day-5. Secondary endpoints were frequency of clinic re-attendance; persistence of presenting problems at follow-up, and proportion of unnecessary antimalarial treatment.

The study was performed in an urban comprehensive health centre in Abeokuta-south district, Ogun state, South-western Nigeria. The health centre is located in the rain forest belt of Nigeria, an area with perennial malaria transmission.

Inclusion criteria were; age 2 – 59months, clinical (IMCI) malaria (defined as axillary temperature of ≥37.5°C or a history of fever within 48hours of presentation) with clinical (IMCI) pneumonia (defined as a presence/history of cough and difficult breathing along with clinician counting of respiratory rate above IMCI cut-offs - a rate of 40 per-minute for children aged 12–59months and above 50 per-minute for children 2–12months), living within 3 kilometers of the health centre, and provision of informed consent. Exclusion criteria were; severe clinical state requiring urgent attention; presence of co-morbidities like otitis media, persistent vomiting, diarrhea etc.

The children were examined by a study clinician and their axillary temperature was measured. Capillary blood was obtained and malaria rapid diagnostic test was performed by a laboratory technician using *Paracheck Pf (Orchid Biomedical Systems, India)*. In the control arm, all the study children received oral antimalarial (artesunate - amodiaquine combination antimalarials) and antibiotic (amoxicillin) irrespective of RDT result. And in the intervention arm, children with a positive RDT result received both antimalarial and antibiotics while those with a negative RDT result received antibiotic only. The artesunate - amodiaquine combination antimalarials used was a 3-day regimen pre-packaged for children 2–11 months or 12–59 months. The oral amoxicillin (antibiotics) used was prescribed at 50mg/kg/day in two divided doses for five days.

All data were recorded in a standard IMCI forms and a follow-up was performed for all patients at day 5. Patients were instructed to return for assessment at any time if the symptoms deteriorated during or after the day-5 follow-up period. Patients who failed to visit the hospital were actively followed - up on day - 6.

Data were double-entered and analyzed with Epi Info (CDC Atlanta, version 3.4.1). Continuous variables were expressed as median (±standard deviation) and categorical variables summarized using percentiles. Group comparisons were made (where appropriate) using the Wilcoxon two-sample test and uncorrected chi-square test, and their corresponding P-values were used for statistical inference.

Before data collection, the Research Committee - Primary Health Care and Disease Control department of Abeokuta-south local government area, Ogun state, Nigeria read and approved the study protocol. Verbal informed consent was obtained from parents/legal guardian of enrolled children.

## Results

The children in the *control and intervention arms* did not differ significantly in their demographic and clinical characteristics (Table 1). Overall, only 46% (95% confidence interval (CI); 36% – 56 %) of the study children had a positive malaria test using RDT. A cure rate of 98% was recorded in the control arm versus 94% in the intervention arm (P=0.31) (Table 2).

**Table 1:** Comparison of two treatment groups at enrollment in Abeokuta-south district, Ogun Nov. 2009 – Feb. 2010.

Variable	Control arm (Presumptive)	Intervention arm (RDT-based)	*Wilcoxon/ Chi-square	P - value
Total enrolled	50	50		
Age (months)				
≤ 12	33 (66%)	31 (62%)	0.17	0.68
> 12	17 (34%)	19 (38%)		
Median (± SD)	12 (± 11.2)	12 (± 9.4)	*1.6	0.2
Gender				
Male	27 (54%)	25 (50%)	0.16	0.69
Female	23 (46%)	25 (50%)		
Axillary temperature				
Range	36.5 – 39.2	36.7 – 39.8		
Median (± SD)	37.9 (± 0.79)	38 (± 0.6)	*0.8	0.77
Malaria rapid diagnostic test				
Positive	22 (44%)	24 (48%)	0.16	0.69
Negative	28 (56%)	26 (52%)		

P -value ≤0.05 is significant, SD = Standard deviation, *Wilcoxon = based on Wilcoxon two sample test;

**Table 2:** Clinical outcome at follow-up (day - 5) in the two groups in Abeokuta-South district, Ogun, Nov. 2009 – Feb. 2010.

Variable	Control arm (Presumptive)	Intervention arm (RDT-based)	*Wilcoxon/Chi-square	P - value
Clinical cure	49/50 (98%)	47/50 (94%)	1.04	0.31
Early clinic re-attendance	3/50 (6%)	9/50 (18%)	3.4	0.065
Fever clearance time (days)				
Range	2 –4	2 –5		
Median (± SD)	2 (± 0.65)	3 (± 0.7)	*32.1	<0.001
Proportion given antimalarial	50/50 (100%)	24/50 (48%)	35.1	<0.001
Persistence of symptoms	1/50 (2%)	3/50 (6%)	1.04	0.31

P-value ≤0.05 is significant, SD = St andard deviation, *Wilcoxon = based on Wilcoxon two sample test;

No death or severe complications were recorded in either group at day-5 follow-up. Median fever clearance time was lower in the *control arm* (2± 0.65 days) than in the *intervention arm* (3± 0.7 days) (P = <0.001). However, rapid diagnostic test -based treated children had lower risk of receiving antimalarials compared to those treated presumptively (*48% vs. 100%)*, (P = <0.001; relative risk 2.08, CI 1.56 to 2.78). Three children in the *intervention arm* versus one child in the *control arm* had persistence of symptoms during follow-up *(P = 0.31).* A repeat RDT for all of them was negative and they were all referred to a tertiary hospital (Table 2).

## Discussion

This study showed that more than half of under-five children with malaria - pneumonia overlap do not have malaria parasitaemia using RDT. Thus, more than half of all antimalarials prescribed presumptively to children with overlap were unnecessary. This finding agrees with that of a Tanzanian study which showed that 43% of the fever in children was caused by acute respiratory infection (ARI) (9). However, it is not consistent with a recent Cameroonian study which showed that ARI accounted for 25.5% of fevers in children 0 – 2 years old (10). These observations suggest that presumptive management of malaria - pneumonia overlap in children is associated with antimalarial drug wastage. Also, it risks the children to be exposed to unnecessary adverse drug reactions like; gastro-intestinal disturbances, neutropenia, hepatotoxicity and neurotoxicity which may be associated with artemisinin - based therapies (11).

After decades of presumptive treatment strategies, the restriction of antimalarials to parasitologically-confirmed cases is necessarily difficult (12). Although recent studies have demonstrated the safety of a RDT-based strategy for children below five years (5, 6, 10, 12), such studies were conducted in the context of fevers in children. We demonstrate that the clinical cure rate of children with malaria - pneumonia overlap treated based on RDT results is not inferior to those managed presumptively. Thus, RDT-based strategy may be considered safe in the management of children with malaria - pneumonia overlap.

A major drawback of the RDT - based strategy in the management of fevers in low resource settings is healthcare providers' failure to adhere to not prescribing antimalarials to children with a negative test (6, 10, 12). This may be experienced more so in children who had malaria - pneumonia overlap because such children will be brought to the hospital in a clinical state enough to cause anxiety amongst their caretakers for they might have attempted home management of the fever as malaria. Although adopting RDT with targeted training of health professionals cannot be assumed to translate adherence to treatment based on test results, strenuous efforts will be needed to understand, and change, existing clinical behavior where healthcare professionals have been used to ignoring negative malaria tests (12).

Children managed based on RDT had a higher median fever clearance time, and re-attended clinic more during follow-up compared to those managed presumptively. This is not consistent with observations of the RDT strategy made in the context of fevers in children (6, 10). The reason/s why a higher proportion of RDT-based managed children had a higher median fever clearance time compared to those managed presumptively are not clear. The implication of late fever clearance in patient treated RDT-based is that caretakers of such children are more likely to re-attend the clinic early and healthcare workers may be tempted to prescribe antimalarials despite a negative RDT result.

This is a pilot study; therefore, the findings are at best preliminary and need to be interpreted with caution. The study suffers from small sample size and lack of randomization, and there may be error from confounding factors from care seeking practices, patient's socio-economic status and/or selection/observer bias. With these limitations, the findings support the rationale for the use of RDTs in the management of children with malaria-pneumonia overlap. A randomized controlled trial that has accommodated these limitations is required to confirm these findings.
